# Personalized Medicine in Psychiatry: From Promise to Practice

**DOI:** 10.3390/jpm16020121

**Published:** 2026-02-18

**Authors:** Gniewko Więckiewicz

**Affiliations:** Department of Psychiatry, Faculty of Medical Sciences in Zabrze, Medical University of Silesia, Pyskowicka 47-51, 42-612 Tarnowskie Góry, Poland; gniewkowieckiewicz@gmail.com

## 1. Personalized Medicine in Psychiatry: From Promise to Practice

The aspiration to personalize psychiatric care has long accompanied the field’s scientific development, yet its realization has often lagged behind advances seen in other areas of medicine [1,2,3]. Psychiatric disorders are heterogeneous, multifactorial, and deeply embedded in biological, psychological, social, and cultural contexts. As a result, the translation of precision and personalized medicine concepts into psychiatry has required not only technological innovation, but also conceptual shifts in how mental illness is defined, measured, and treated. This Special Issue, “Personalized Medicine in Psychiatry: Challenges and Opportunities”, was conceived to capture this moment of transition: one in which psychiatry is moving beyond uniform treatment algorithms toward stratified, data-informed, and person-centered approaches.

The contributions assembled here reflect the breadth of this transformation. Together, they illustrate how personalized psychiatry is no longer anchored to a single domain—such as genetics or pharmacology—but is instead emerging from the convergence of systems neuroscience, clinical epidemiology, psychotherapeutic science, digital health, public policy, and patient experience. Rather than offering a definitive blueprint, this Special Issue provides a multidimensional snapshot of where the field currently stands, what it has learned, and what remains unresolved.

## 2. Rethinking Heterogeneity: From Diagnoses to Dynamic Profiles

A recurring theme across this collection is the recognition that diagnostic categories alone are insufficient to guide personalized care. Several contributions challenge the assumption that individuals who share a diagnosis necessarily share mechanisms, symptom dynamics, or treatment needs. Instead, they highlight how symptom-level architectures, residual symptom patterns, and longitudinal trajectories are more important and informative units of personalization [4,5].

Network-based and system-level approaches exemplify this shift. By modeling psychiatric symptoms as interacting systems rather than isolated outcomes, such methods reveal how different biological or contextual factors can reshape psychopathology from within. The observation that depressive symptom networks differ according to metabolic status underscores the need to integrate somatic health into psychiatric stratification (contribution 1). Fatigue, sleep, and appetite disturbances emerge not merely as secondary symptoms, but as central nodes in specific subgroups, suggesting that personalization may depend as much on physiological context as on psychiatric diagnosis (contribution 2).

Complementing this perspective, narrative and systematic reviews addressing residual symptoms and treatment resistance emphasize how incomplete remission continues to impair functioning and predict relapse. Importantly, they expose inconsistencies in how residual symptoms are defined, measured, and prioritized. These inconsistencies are not trivial: they shape clinical decisions, research outcomes, and patient expectations. The contrast between clinician-centered endpoints and patient-valued outcomes—such as well-being, vitality, and positive affect—highlights a persistent gap that personalized medicine must bridge if it is to be clinically meaningful.

## 3. Treatment Resistance as a Lens on Personalization

Treatment resistance occupies a central position in discussions of personalized psychiatry, not only as a clinical challenge but also as a conceptual stress test for existing models of care [6]. Several contributions approach resistance from complementary angles, illustrating that it cannot be reduced to pharmacological failure alone.

Quantitative syntheses of psychotherapy in treatment-resistant depression demonstrate that psychological interventions retain efficacy even after multiple treatment failures, albeit with modest effect sizes and substantial heterogeneity (contribution 3). These findings reinforce the value of multimodal care while simultaneously exposing the limits of current evidence. The scarcity of trials tailored specifically to resistant populations suggests that personalization remains more aspirational than operational in this domain [7].

At the other end of the evidentiary spectrum, psychodynamically informed case analyses remind us that treatment resistance often unfolds within relational contexts (contribution 4). Patient–physician dynamics, trust, continuity, and emotional histories can decisively shape treatment adherence and outcomes. These qualitative insights challenge technologically driven visions of personalized medicine by underscoring that personalization is not solely a matter of selecting the “right” intervention, but also a matter of sustaining therapeutic relationships capable of supporting change.

Together, these perspectives suggest that treatment resistance is best understood as an emergent property of interacting biological, psychological, and interpersonal factors. Addressing it requires flexibility not only in treatment selection, but also in clinical reasoning, care structures, and expectations of recovery.

## 4. Expanding the Therapeutic Landscape

Personalized psychiatry also depends on expanding the range of available interventions and understanding for whom, and under what conditions, they are most effective. The contributions in this Special Issue reflect a growing openness to both novel and non-traditional approaches, while maintaining a commitment to methodological rigor and ethical responsibility.

Interdisciplinary reviews of psychedelic research exemplify this balance [8,9]. By situating psychedelics within psychological, neuroscientific, anthropological, and philosophical frameworks, such work reframes these compounds not as isolated pharmacological agents, but as catalysts of experiential and neuroplastic change. Their potential therapeutic value lies not only in symptom reduction, but also in their capacity to disrupt rigid cognitive and emotional patterns—an effect that may be particularly relevant for treatment-resistant conditions. At the same time, calls for ethical safeguards and rigorous research designs caution against premature clinical enthusiasm (contribution 5).

Non-pharmacological interventions are receiving parallel attention. Preclinical studies demonstrating that physical exercise modulates brain metabolism following chronic substance exposure provide biological grounding for lifestyle-based personalization strategies (contribution 6). Similarly, randomized trials of brief, app-based contemplative interventions reveal that digital scalability does not guarantee universal benefit (contribution 7). Instead, individual differences in stress reactivity and resilience appear to determine who gains from low-intensity interventions and who requires more intensive, personalized support.

These findings converge on a critical insight: personalization is not only about choosing between treatments, but also about matching intervention intensity, modality, and delivery format to individual capacities for plasticity and recovery.

## 5. From Evidence to Implementation: Systems Matter

Even the most sophisticated personalized approaches remain aspirational if they cannot be implemented within real-world health systems. Several contributions therefore turn their attention to the infrastructural and policy dimensions of personalized psychiatry [10].

Analyses of payer coverage decisions for pharmacogenomic testing reveal that access to personalized tools is shaped less by the absence of evidence and more by how evidence is interpreted and valued (contribution 8). Real-world evidence, case series, and observational studies play a significant role in these decisions, yet their influence varies across payer types and policy contexts. This variability underscores the need for clearer standards and dialog between researchers, clinicians, and policymakers regarding what constitutes actionable evidence in personalized care.

Digital health and informatics further extend the scope of personalization from individual encounters to population-level strategies. Electronic health records, decision support systems, prescription monitoring programs, and artificial intelligence-driven analytics offer unprecedented opportunities to identify risk, guide interventions, and reduce inequities in access. At the same time, they raise questions about data quality, privacy, algorithmic bias, and the balance between automation and clinical judgment. In this sense, personalized psychiatry becomes as much a matter of governance and ethics as of innovation (contribution 9).

## 6. Challenges, Opportunities, and the Road Ahead

Taken together, the contributions to this Special Issue suggest that personalized medicine in psychiatry is no longer a distant horizon, but neither is it a finished paradigm. Its most promising advances arise not from any single technology or discipline, but from their integration. Biological markers, symptom networks, psychological processes, digital tools, and relational dynamics each illuminate different facets of mental illness [11]. The challenge lies in weaving these perspectives into coherent, flexible models of care.

Several priorities emerge for future research and practice. First, greater conceptual clarity is needed regarding key constructs such as residual symptoms, treatment resistance, and remission. Without shared definitions, personalization risks becoming fragmented and inconsistent. Second, stratification efforts must move beyond cross-sectional markers to incorporate longitudinal trajectories of vulnerability, resilience, and change. Third, implementation science must be elevated to the same status as discovery science, ensuring that personalized tools are accessible, equitable, and responsive to real-world constraints (contribution 10).

Finally, personalization should not be conflated with complexity for its own sake. The ultimate measure of success lies in whether personalized approaches improve outcomes that matter to patients: functioning, well-being, dignity, and sustained recovery. Achieving this goal will require not only innovation, but also humility—an openness to revising assumptions, integrating diverse forms of evidence, and centering the lived experience of those whom psychiatry seeks to serve.

This Special Issue does not the conversation on personalized medicine in psychiatry to a close; rather, it clarifies its contours and stakes. We hope it will serve as both a reference point and a catalyst, encouraging continued interdisciplinary collaboration as the field moves from promise to practice.
